# Adipose triglyceride lipase driven lipolysis as a targetable metabolic vulnerability in prostate cancer with intrinsic metabolic inflexibility

**DOI:** 10.1038/s41416-026-03545-4

**Published:** 2026-07-23

**Authors:** Andreas Tiefenbacher, Madeleine Gudenus, Daniel Valcanover, Barbara Neudert, Raheleh Sheibani-Tezerji, Theresa Mendrina, Jessica Kalla, Laura Pajed, Kristina Draganić, Andrea Haitel, Astrid Haase, Markus Hartenbach, Sazan Rasul, Walter Berger, Rolf Breinbauer, Gernot Grabner, Martina Schweiger, Eva Compérat, Gerda Egger

**Affiliations:** 1 Department of Pathology, Medical University of Vienna, Vienna, Austria; 2 Comprehensive Cancer Center, Medical University of Vienna, Vienna, Austria; 3 Center for Cancer Research, Medical University of Vienna, Vienna, Austria; 4 Institute of Inorganic Chemistry, Faculty of Chemistry, University of Vienna, Vienna, Austria; 5 Institute of Molecular Biosciences, University of Graz, Graz, Austria; 6 Department of Biomedical Imaging and Image-guided Therapy, Division of Nuclear Medicine, Medical University of Vienna, Vienna, Austria; 7 Institute of Organic Chemistry, Graz University of Technology, Graz, Austria; 8 Gottfried Schatz Research Center, Medical University of Graz, Graz, Austria; 9 BioTechMed-Graz, Graz, Austria

**Keywords:** Cancer metabolism, Prostate cancer, Targeted therapies

## Abstract

**Background:**

Despite the widespread clinical use of prostate-specific membrane antigen (PSMA)-targeted imaging and therapy in prostate cancer, the biological functions underlying PSMA-associated tumour aggressiveness remain incompletely understood.

**Methods:**

PSMA-PET-guided sampling of paired PSMA-high and PSMA-low tumour regions was used for integrated proteomic and epigenomic analyses. PSMA-ATGL correlation was validated by immunohistochemistry in 90 treatment-naïve patients. Metabolic profiling was performed using Seahorse Mito Fuel Flex Test assays under unperturbed conditions and pharmacological or genetic perturbation of ATGL and PSMA. Therapeutic vulnerability was assessed using NG-497 and siRNA-mediated knockdown.

**Results:**

PSMA-high tumours exhibited lipolytic reprogramming characterised by ATGL upregulation, confirmed by a robust PSMA-ATGL correlation in the validation cohort (*P* < 0.0001). Despite shared fatty acid dependency, LNCaP cells exhibited intrinsic metabolic inflexibility while 22RV1 cells displayed high adaptability. Both pharmacological and genetic ATGL inhibition most potently impaired LNCaP proliferation. Enzalutamide upregulated ATGL in both cell lines, revealing reciprocal regulation between AR signaling and the PSMA/ATGL axis.

**Conclusions:**

ATGL-mediated lipolysis represents a promising therapeutic target in prostate cancer, with heightened sensitivity in tumours lacking metabolic flexibility to utilize alternative pathways.

## Background

Prostate-specific membrane antigen (PSMA) is a clinically validated biomarker in prostate cancer, widely employed in nuclear medicine for Positron Emission Tomography (PET) imaging and targeted ^177^Lu radioligand therapy [1–3]. Elevated PSMA expression correlates with aggressive disease features, including higher histological grade and advanced tumour stage, and serves as a predictor of biochemical recurrence [4–6]. Notably, PSMA-PET imaging frequently reveals substantial intratumor heterogeneity [7], posing challenges for accurate disease stratification [8] and therapeutic efficacy [7, 9]. In prostate cancer, PSMA was shown to regulate the PI3K-Akt signalling axis, following PSMA-dependent cleavage of glutamated folate and subsequent activation of the metabotropic glutamate receptors [10]. Furthermore, PSMA interacts with the scaffolding protein RACK1, thereby disrupting the IGF-1R/RACK1/β1-integrin complex and directing signalling towards PI3K-Akt pathways [11].

A fundamental characteristic of cancer is the reprogramming of energy metabolism, thereby creating potential vulnerabilities for therapeutic intervention [12, 13]. In prostate cancer, metabolic adaptations include increased lipolysis, which provides energy and building blocks for tumour growth and progression [14]. Adipose triglyceride lipase (ATGL), encoded by the *PNPLA2* gene, is the rate-limiting enzyme in the initial step of neutral lipolysis, hydrolysing triglycerides into diglycerides and fatty acids in the cytoplasm [15]. Despite its established role in lipolysis in adipocytes, the precise impact of ATGL on cancer progression remains unclear, with published reports demonstrating variable effects ranging from tumour-suppressive [16–19] to tumour-promoting [20–22] effects. These contradictory findings indicate that the factors determining ATGL function vary across cancer contexts and require further investigation.

Here, we identified a strong correlation between PSMA and ATGL protein expression in primary prostate tumours and characterised the metabolic landscape of prostate cancer cell lines with distinct PSMA and ATGL expression profiles. Using the human-specific ATGL inhibitor NG-497 [23], we demonstrate that sensitivity to ATGL inhibition is not determined by target expression alone but by intrinsic metabolic inflexibility. These findings suggest that metabolic profiling in combination with target expression analysis may serve as a framework for identifying patients who would benefit from anti-ATGL therapies.

## Results

### Biological profiling of PSMA expression reveals lipolytic metabolic reprogramming in prostate cancer

To investigate the biological role of PSMA in prostate cancer, we initially performed in silico analyses of publicly available TCGA-PRAD transcriptomics data [24], stratifying tumour samples (*n* = 497) based on the expression levels of *FOLH1*, encoding PSMA, into high and low groups (Supplementary Fig. S1a). Differential gene expression analysis revealed hierarchical clustering of *FOLH1* high and low groups into two distinct entities (Supplementary Fig. S1b). Significant alterations in the transcriptome associated with high *FOLH1* expression were identified for 652 differentially expressed genes (240 upregulated, 412 downregulated). Functional enrichment analysis of these genes uncovered significant gene set enrichment in a variety of pathways related to cell adhesion and signalling (Supplementary Fig. S1c). Notably, “Fatty Acid Oxidation” exhibited the highest enrichment ratio, suggesting a potential metabolic reprogramming linked to high *FOLH1* expression in prostate cancer.

Due to the involvement of the PSMA substrate folate as an essential co-factor in the one-carbon metabolism, which is the basis for the generation of the methyl-donor *S*-adenosylmethionine, we next examined DNA methylation profiles in the same tumour subsets. This revealed a significantly elevated global methylation level in the *FOLH1*-high group (Supplementary Fig. S1d), with 661 specific CpG sites exhibiting differential methylation (Supplementary Fig. S1e). Together, these findings suggest that high *FOLH1* expression in prostate cancer is associated with both significant transcriptional and epigenetic reprogramming.

**Fig. 1:**
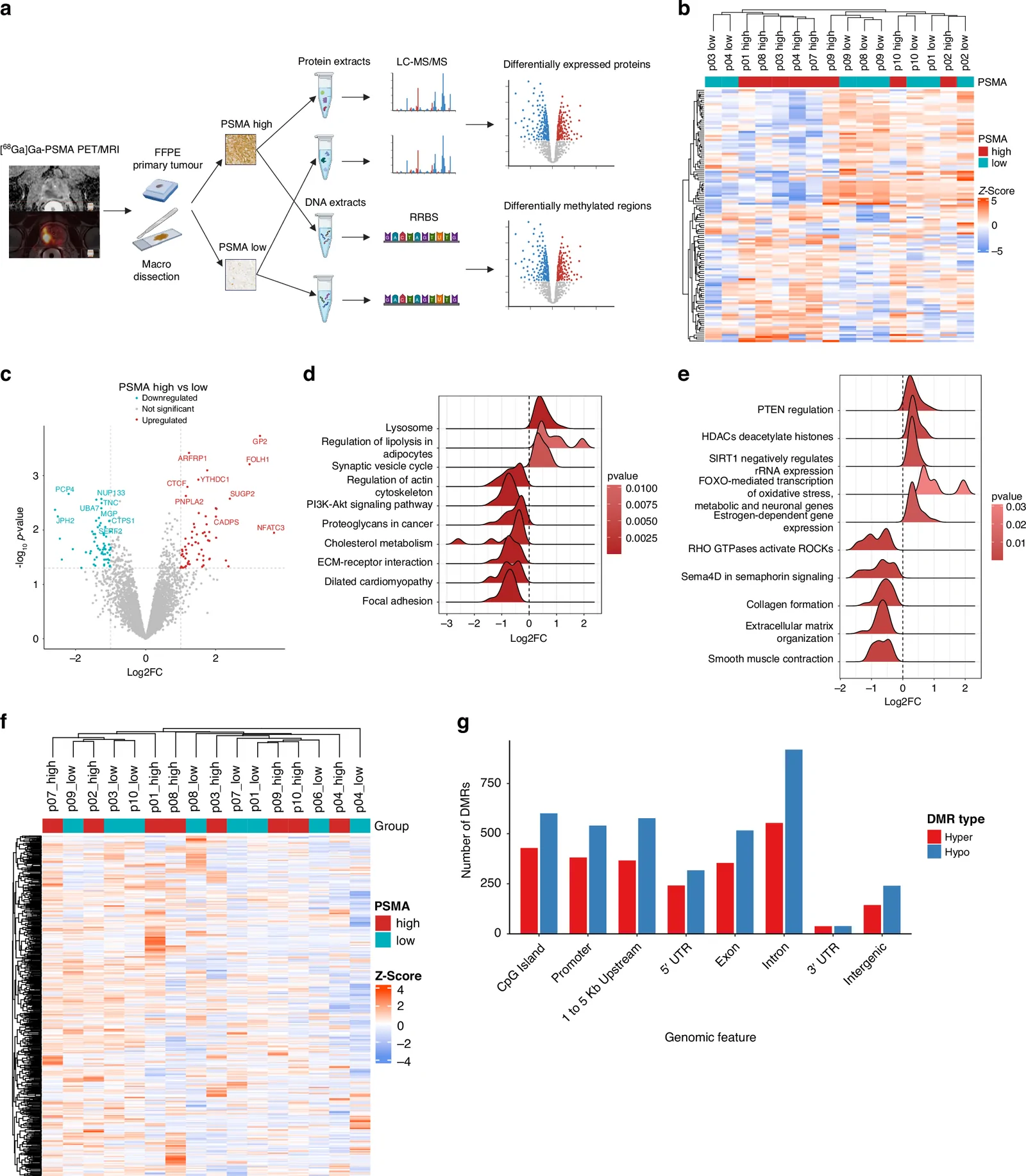
Proteomic and epigenetic profiling of differential PSMA-associated prostate tumor areas. **a** Experimental workflow for proteomic and DNA methylation analysis of patient-matched PSMA-high and -low prostate tumor areas. **b** Unsupervised clustering of significantly differentially expressed proteins (DEPs; absolute Log2FC > 1, *P* < 0.05) in PSMA-high vs. PSMA-low tumor areas. The heatmap represents Z-score normalized values with blue indicating low and red indicating high expression values. **c** Volcano plot of DEPs. Red points indicate significantly upregulated proteins in PSMA-high areas; blue points indicate significantly downregulated proteins (absolute Log2FC > 1, *P* < 0.05). Ridge plots visualizing the distribution of absolute expression Log2FC (*x*-axis) for core-enriched proteins within selected KEGG (**d**) and REACTOME (**e**) pathways identified by GSEA. The shape of each ridge represents the density distribution, indicating the frequency of proteins at specific Log2FC values within a given pathway. **f** Unsupervised hierarchical clustering of the top 1000 differentially methylated CpGs of PSMA-high and -low tumor areas identified by reduced representation bisulfite sequencing (RRBS). The heatmap represents Z-score normalised methylation values with red indicating hypermethylated and blue hypomethylated CpGs. **g** Distribution of differentially methylated regions (DMRs) according to genomic annotations between PSMA-high and low tumor areas. Bars represent the number of hypermethylated (red) and hypomethylated (blue) DMRs identified across various genomic features as indicated on the *x*-axis.

To extend these findings through exploratory proteomic and epigenetic profiling in our own patient cohort, we utilised radical prostatectomy specimens from 10 patients with locally advanced prostate cancer (Gleason score 8 and 9) and lymph node involvement (Supplementary Table 1). Patient selection was guided by retrospective analysis of [^68^Ga]Ga-PSMA-PET imaging data of patients with heterogenous PSMA-PET signals, indicative of high and low PSMA-expressing areas within their primary prostate tumour. Label-free liquid chromatography-tandem mass spectrometry (LC-MS/MS) and reduced representation bisulfite sequencing (RRBS) were then performed to compare protein expression and DNA methylation of PSMA-high versus PSMA-low areas from within the same specimen, determined by PSMA immunohistochemistry (IHC) (Fig. 1a). In two out of ten cases, we were unable to confidently identify areas of low PSMA expression by IHC these samples were excluded from the proteomics analysis. The proteomics approach identified 4473 proteins across all samples, with 3109 proteins shared between all samples analysed (Supplementary Fig. S2a). Differential expression analysis revealed 127 significantly differentially regulated proteins (*P* < 0.05, absolute Log2FC > 1), with PSMA (FOLH1) among the top deregulated proteins, validating our approach (Fig. 1b, c, and Supplementary Table 3). Gene Set Enrichment Analysis (GSEA) highlighted a significant upregulation of lysosomal and vesicle-associated KEGG pathways as well as lipolysis in PSMA-high areas, corroborating our in silico findings (Fig. 1d). Moreover, both KEGG and REACTOME pathway analysis highlighted a downregulation of genes associated with extracellular matrix (ECM) organisation and collagen formation (Fig. 1d, e). Notably, a deregulation of PTEN regulation and PI3K-Akt signalling was evident in PSMA-high tumour areas, alongside other deregulated transcriptional pathways. Together, these data suggest that PSMA-high tumour areas show differences in their metabolic characteristics, ECM remodelling and tumour signalling compared to PSMA-low areas within the same patient.

For DNA methylation analysis based on RRBS, quality control filtering due to insufficient DNA yield led to the exclusion of three samples. In line with TCGA-PRAD methylation data analysis, PSMA-high samples exhibited a trend towards higher global DNA methylation levels, although this was not statistically significant (*P* = 0.65; Supplementary Fig. S2c). Hierarchical clustering based on the top 1000 differentially methylated loci (DML) did not reveal a clear separation between PSMA-high and -low tumour areas (Fig. 1f). However, differentially methylated region (DMR) analysis identified 2238 DMRs between the PSMA-high and low groups (Supplementary Table 4). Analysis of the genomic distribution of these DMRs revealed a predominant enrichment within promoter regions including 1–5 kb upstream of the transcription start site and intronic regions (Fig. 1g).

### Integrated proteomic and epigenomic analysis identifies ATGL as a key PSMA-associated lipolytic enzyme

**Fig. 2:**
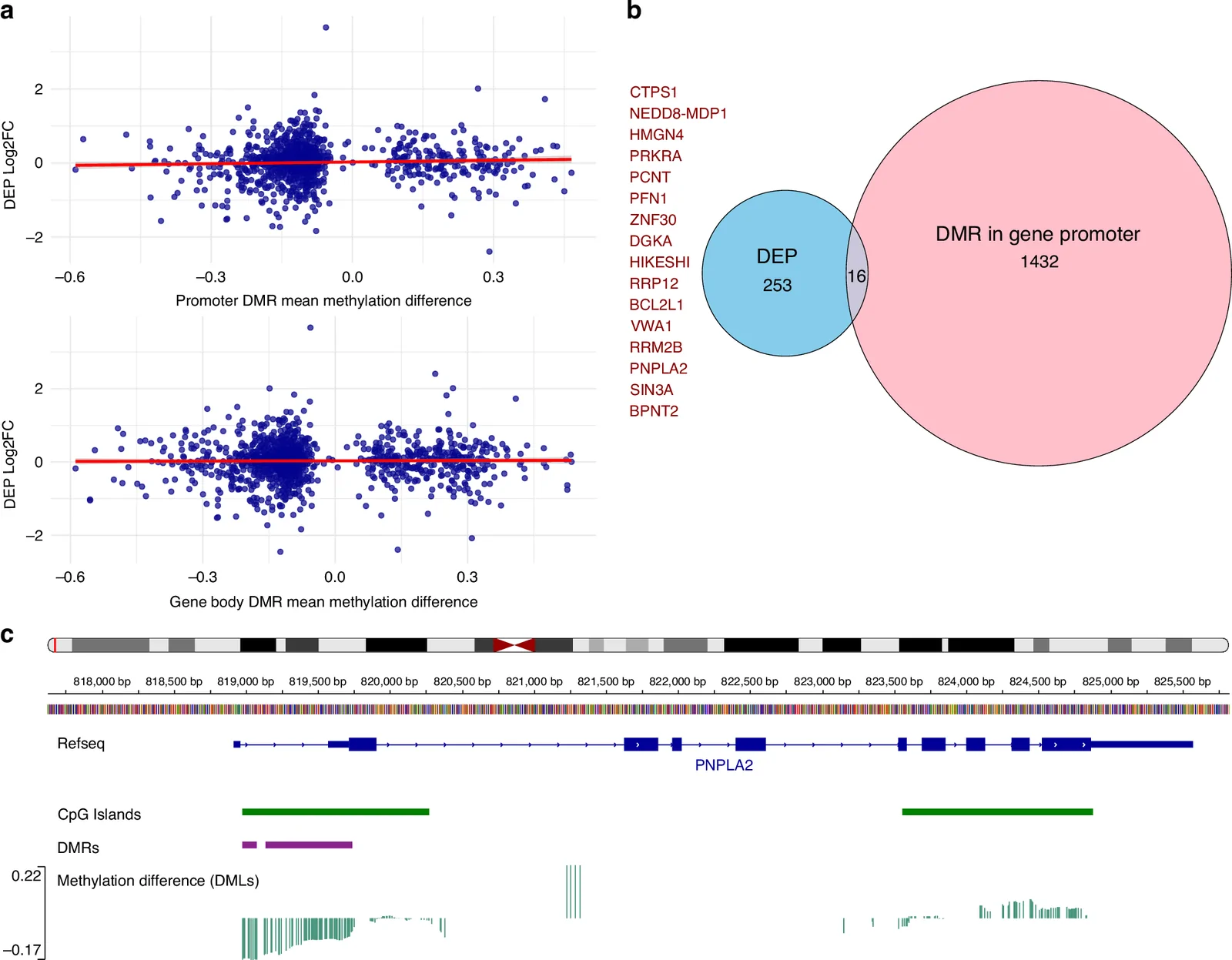
Integrative analysis highlights ATGL upregulation and promoter hypomethylation in PSMA-high prostate cancer. **a** Scatter plots showing the absence of significant correlations between protein expression changes and DNA methylation levels within promoters (top) or gene bodies (bottom) of differentially expressed proteins (DEP). **b** Venn diagram illustrating the overlap between significantly differentially expressed proteins (*P* < 0.05) and genes harboring promoter-associated differentially methylated regions (DMRs), highlighting 16 overlapping proteins. **c** UCSC genome browser view of the *PNPLA2* locus, encoding for ATGL protein. Tracks show Refseq gene annotation (blue), CpG islands downloaded from UCSC (green), DMRs (purple), and *β*-value differences for individual CpG sites (DMLs, turquoise).

To further refine our focus on biologically relevant proteins and identify those with convergent epigenetic regulation, we integrated our proteomics and RRBS datasets. Initial correlation analysis between protein expression changes and DMR methylation within gene bodies or promoters yielded no significant associations (Fig. 2a). However, by identifying the overlap between significantly differentially expressed proteins (*P* < 0.05) and genes harbouring promoter-associated DMRs, we pinpointed 16 candidate proteins (Fig. 2b). This list included ATGL, encoded by the *PNPLA2* gene. Notably, the *PNPLA2* locus showed a clear hypomethylation pattern in its promoter CpG island in PSMA-high samples, providing epigenetic support for ATGL protein overexpression (Fig. 2c).

### Independent cohort validates strong PSMA-ATGL correlation and identifies links to clinicopathological and metabolic features

**Fig. 3:**
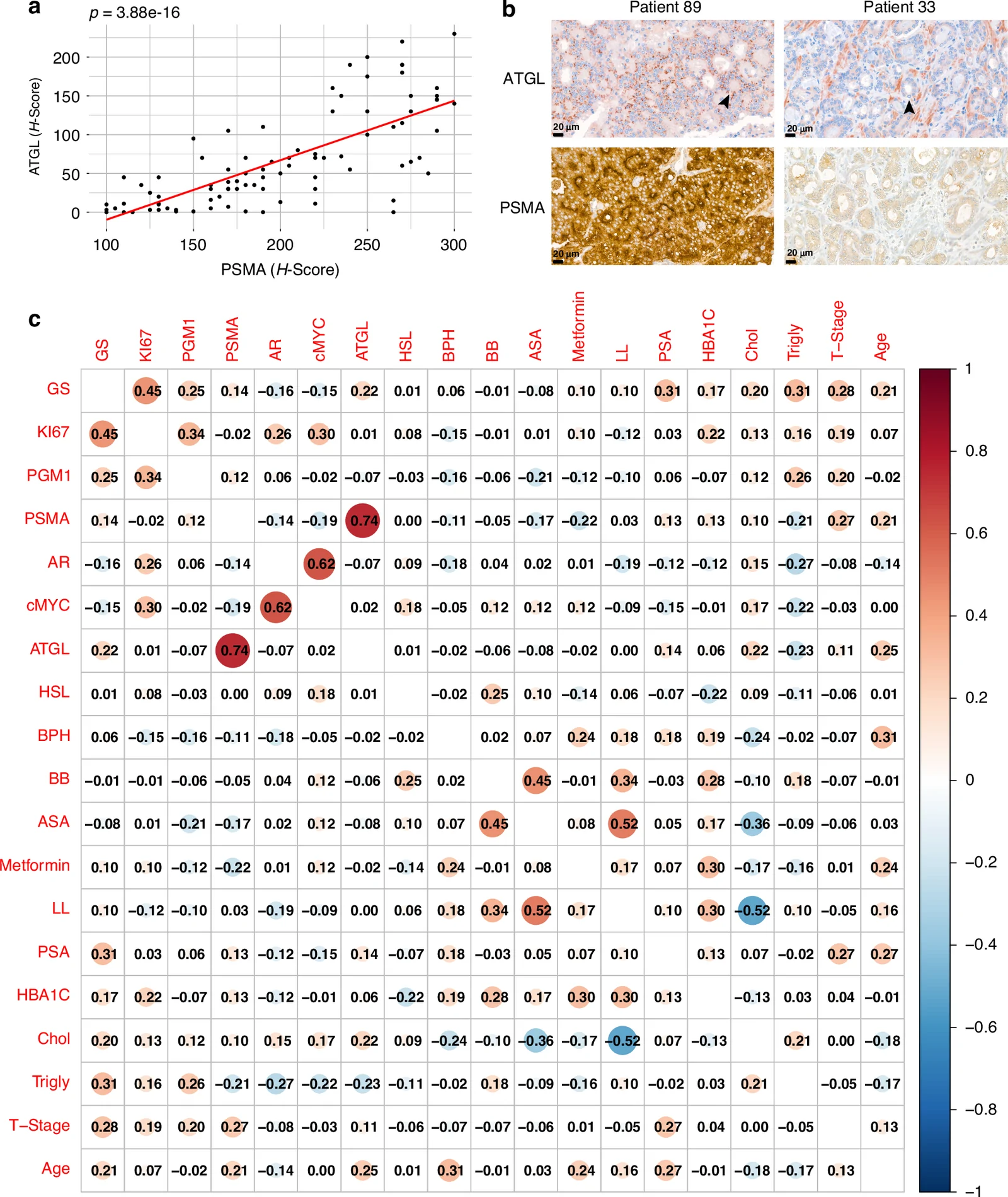
Validation of PSMA-ATGL protein co-expression and associations with clinicopathological and biological markers in an independent prostate cancer cohort. **a** Scatter plot and linear regression line depicting the robust positive correlation between ATGL and PSMA protein expression H-scores in the tissue micro-array (TMA) cohort (*n* = 90). **b** Representative IHC staining images illustrating high (left, patient 89) and low (right, patient 33) ATGL (top) and PSMA (bottom) protein expression in TMA cores. Black arrowheads indicate representative tumour glands with strong specific (left) and negative (right) ATGL stain. **c** Heatmap visualizing the Pearson correlation matrix for all investigated variables in the TMA cohort. Color scale indicates the Pearson correlation coefficient, ranging from strong positive (red) to strong negative (blue). Circle size indicates absolute strength of correlation. GS (Gleason Score), BPH (benign prostate hyperplasia medication e.g. 5-alpha reductase inhibitors), BB (beta blockers), ASA (acetylsalicylic acid), LL (lipid-lowering drugs e.g. statins), HbA1c (glycated haemoglobin - serum marker for average blood glucose levels for the three months prior to prostatectomy), Chol (pre-operative serum cholesterol level), Trigly (pre-operative serum triglyceride level.

To validate the correlation between PSMA and ATGL protein expression in a larger, independent patient cohort, we generated tissue microarrays (TMAs) of treatment-naïve primary prostate cancer samples of intermediate Gleason scores (3+4, 4+3, and 4+4) (*n* = 93; Supplementary Table 2). Due to the absence of tumour tissue in TMA cores from 3 patients, the final analysis included 90 patient samples. PSMA showed a balanced IHC staining score, while ATGL expression was predominantly expressed at lower levels across all samples (Supplementary Fig. S3a, b). However, in line with our proteomics data, we observed a robust positive correlation between ATGL and PSMA protein expression in the TMA cohort (*r* = 0.74, *P* < 0.0001; Fig. 3a, b), validating our initial findings in a clinically relevant patient group.

Next, we correlated PSMA and ATGL protein expression with molecular and clinicopathological patient data including Gleason score and T-stage, metabolic blood markers, or medication history, as well as IHC staining for specific candidate genes (Fig. 3c, and Supplementary Fig. S3c–f). PSMA expression was similar in the three Gleason Score groups, but as expected, showed significantly higher expression in T3 compared to T2 stage tumours (*P* = 0.011) (Supplementary Fig. S3c). On the other hand, ATGL expression was significantly higher in Gleason 4 + 4 tumours compared to Gleason 3+4 tumours (*P* = 0.038), but showed only a tendency of higher expression in T3 stage samples (*P* = 0.347) (Supplementary Fig. S3d).

To explore potential metabolic links, we analysed associations between PSMA and ATGL protein expression and serum metabolic markers. A negative correlation trend was observed between serum triglyceride levels and PSMA expression (*r* = −0.21, *P* = 0.517; Supplementary Fig. S3e) as well as ATGL expression (*r* = −0.27, *P* = 0.473; Supplementary Fig. S3e). Notably, ATGL expression demonstrated a statistically significant positive correlation with serum cholesterol levels (*r* = 0.22, *P* = 0.020; Supplementary Fig. S3f). No associations of PSMA and ATGL expression with different medications, including lipid-lowering or antidiabetic drugs, were detected (Fig. 3c). Moreover, expression levels of the proliferation marker Ki-67, c-MYC or hormone-sensitive lipase (HSL), the second enzyme in the lipolytic cascade with primary diglyceride but also triglyceride hydrolase activity, were unrelated to PSMA and ATGL expression. We also measured PGM1 expression as a surrogate of tumour-associated macrophage infiltration, which was associated with Ki-67 expression, but showed no correlation with PSMA or ATGL. Interestingly, we observed weak positive correlations between age and both PSMA (*r* = 0.21, *P* = 0.026) and ATGL (*r* = 0.25, *P* = 0.012) protein expression levels. Together, these data suggest correlative associations between serum lipid profiles and tumoral ATGL expression, with lower triglyceride and higher cholesterol levels observed in patients with ATGL-high prostate cancer. However, as serum metabolite levels are influenced by numerous systemic factors including diet, lifestyle, and comorbidities that were not controlled for in this retrospective analysis, these associations should not be interpreted as direct surrogates for tumour-intrinsic metabolic activity.

### In vitro metabolic characterization reveals distinct fuel utilization and flexibility profiles

**Fig. 4:**
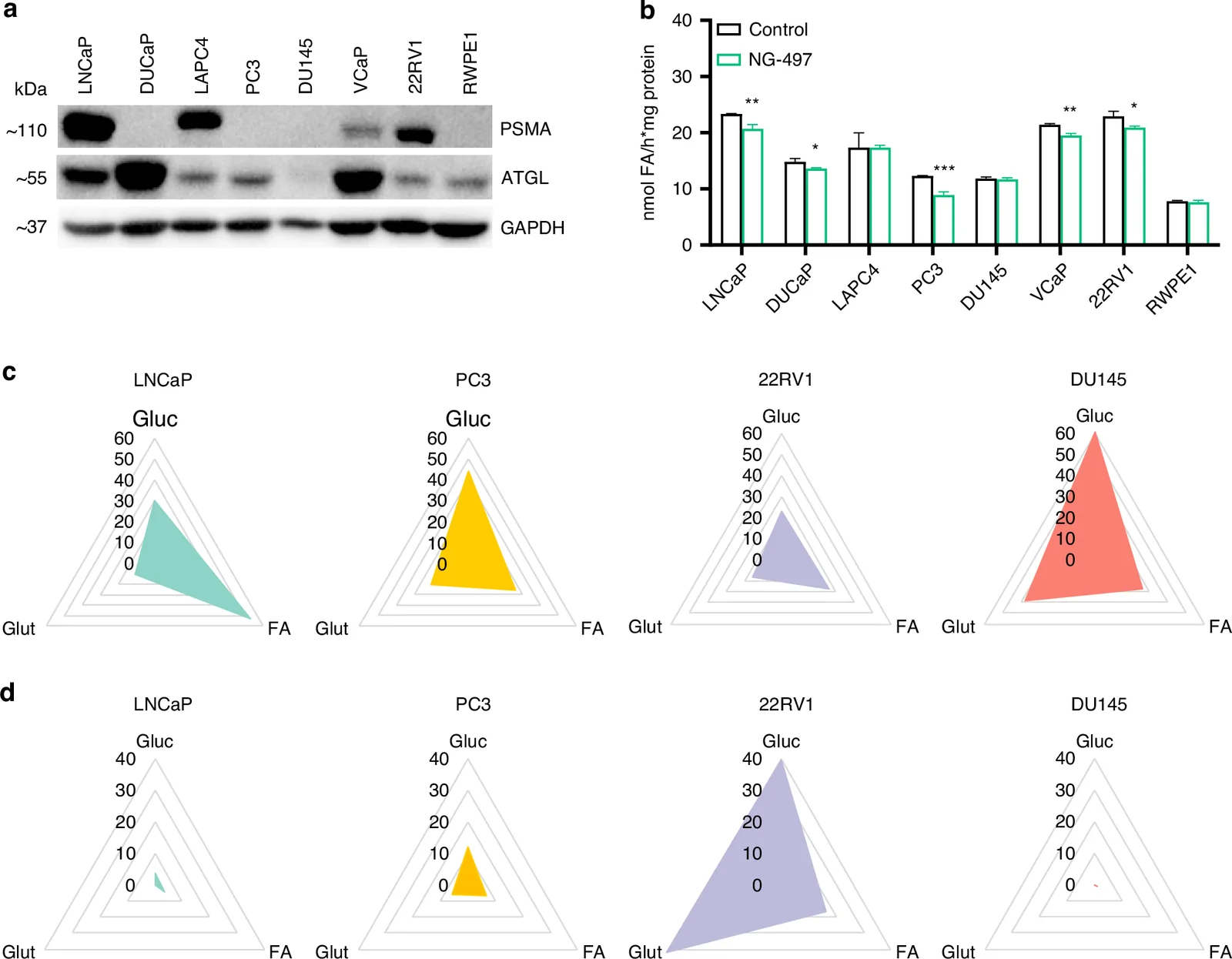
In vitro metabolic profiling of prostate cancer cell lines. **a** Western blot analysis of PSMA and ATGL protein expression across a panel of prostate cancer cell lines and the non-neoplastic prostate epithelial cell line RWPE1. GAPDH was used as a loading control. **b** Triglyceride hydrolase activity assay in prostate cancer and RWPE1 cell lines. Cell lysates were incubated with vehicle control (Control) or 20 µM NG-497 for 1 h (NG-497). Released fatty acids (nmol/hour) per mg of protein are shown. Data represent mean ± SEM (*n* = 3). Statistical significance was determined by unpaired *t*-test (^*^P < 0.05, ^**^P < 0.01, ^***^P < 0.001 vs. vehicle control within each cell line). **c** Relative dependency on glucose (Gluc), glutamine (Glut), and fatty acid (FA) oxidation in selected prostate cancer cell lines as determined by Seahorse XF Mito Fuel Flex Test assay. **d** Fuel flexibility of selected prostate cancer cell lines, representing the percentage maximal compensatory increase in oxygen consumption rate (OCR) upon inhibition of alternative fuel pathways as determined by Seahorse XF Mito Fuel Flex Test assay.

To dissect the functional role of ATGL in prostate cancer in more detail, we sought to identify suitable cell line models by characterising ATGL and PSMA protein expression across a panel of seven prostate cancer cell lines and one non-neoplastic prostate epithelial cell line (RWPE1). Western blot analysis revealed a diverse range of expression levels for both PSMA and ATGL across the cells tested (Fig. 4a). PSMA protein expression was highest in LNCaP, LAPC4, and 22RV1 cells, whereas ATGL protein expression was elevated in LNCaP, DUCaP, and VCaP cells. Notably, this analysis did not reveal a consistent correlation between PSMA and ATGL protein expression levels, probably due to their mainly metastatic origin or their metabolic adaptation in vitro.

To directly assess ATGL enzymatic activity and the impact of its pharmacological inhibition, we employed a triglyceride hydrolase activity assay measuring the release of fatty acids from radioactively labelled triglycerides following incubation with cell lysates. Treatment with the ATGL inhibitor NG-497 resulted in significant inhibition of triglyceride hydrolase activity across multiple prostate cancer cell lines, including LNCaP, VCaP, PC3, 22RV1, and DUCaP cells (*P* < 0.05; Fig. 4b), confirming the efficacy of ATGL inhibition in these models.

Based on these initial findings and considering the combinatorial diversity of PSMA and ATGL expression profiles, we selected four cell lines for subsequent metabolic analyses: LNCaP (PSMA-high, ATGL-high), 22RV1 (PSMA-high, ATGL-low), PC3 (PSMA-negative, ATGL-high), and DU145 (PSMA-negative, ATGL-low). To assess the influence of growth media on ATGL activity and protein expression, we cultured these four cell lines in human plasma like medium (HPLM), which more closely mimics physiological glucose levels compared to standard RPMI-1640 medium (Supplementary Methods Fig. 2). Culturing cells in HPLM did not significantly alter ATGL activity and response to NG-497 (Supplementary Fig. S4a), or PSMA, ATGL, HSL and ABHD5 protein expression levels, although c-MYC protein expression was consistently upregulated in HPLM conditions (Supplementary Fig. S4b).

Next, we quantified the basic metabolic profiles of the selected cell lines, including their fuel dependency and metabolic flexibility using Seahorse XF Mito Fuel Flex Test assays. Analysis of baseline OCR revealed that LNCaP and 22RV1 cells exhibited over twofold higher baseline respiration rates compared to PC3 and DU145 cells (Supplementary Fig. S4c). Fuel dependency analysis indicated that LNCaP and 22RV1 cells exhibited a marked dependency on fatty acid oxidation, with 22RV1 cells displaying a similar dependency on glucose (Fig. 4c). Conversely, PC3 and DU145 cells were primarily dependent on glucose to maintain baseline respiration. Regarding metabolic flexibility, which indicates the capacity of cells to increase the oxidation of one fuel when alternative pathways are inhibited, 22RV1 cells displayed high flexibility across all fuel pathways, suggesting metabolic adaptability (Fig. 4d). In contrast, both LNCaP and DU145 cells showed very limited metabolic flexibility, while PC3 cells exhibited intermediate flexibility primarily towards glucose.

In summary, metabolic profiling of the selected cell lines revealed distinct fuel utilisation patterns, with LNCaP and 22RV1 cells exhibiting high baseline mitochondrial respiration driven by fatty acid oxidation in contrast to the glucose-centric metabolism of PC3 and DU145.

### LNCaP cells exhibit enhanced vulnerability to the ATGL inhibitor NG-497 and ATGL knockdown

**Fig. 5:**
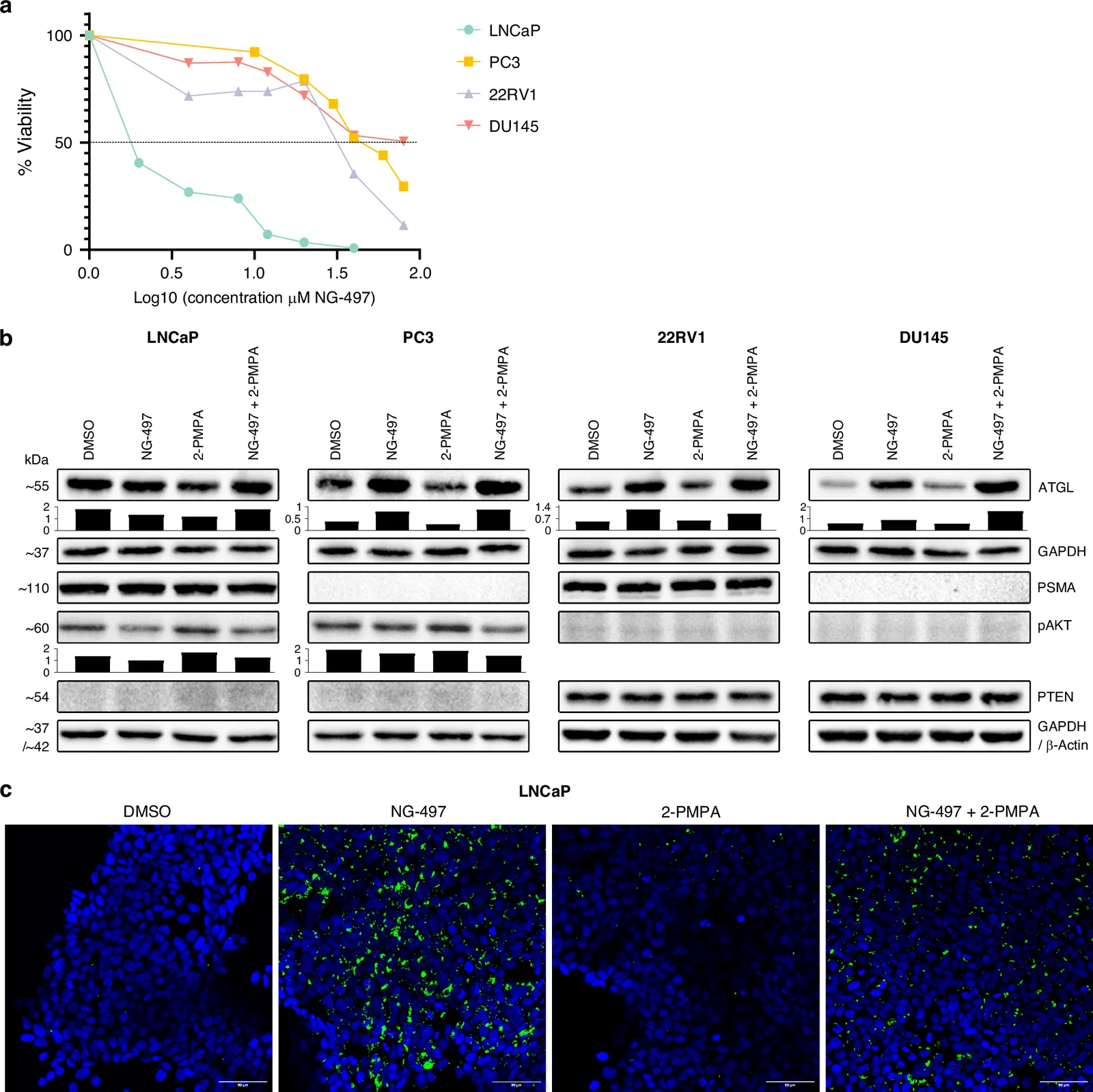
ATGL inhibition by NG-497 selectively impairs viability and induces lipid accumulation. **a** NG-497 dose-viability curves for selected prostate cancer cell lines, as determined by CyQUANT® Cell Proliferation Assay after 72 h of treatment with NG-497. Data represent mean of *n* = 3. IC50 values are indicated by the horizontal line. **b** Western blot analysis showing protein expression levels of ATGL, PSMA, pAKT and PTEN in selected prostate cancer cell lines. Cells were treated with DMSO (vehicle control), IC50 concentration of NG-497 (2,2 µM, 51 µM, 27 µM and 80 µM for LNCaP, PC3, 22RV1 and DU145, respectively), 2-PMPA (50 µM), and NG-497 (2,2 µM, 51 µM, 27 µM and 80 µM for LNcaP, PC3, 22RV1 and DU145, respectively) + 2-PMPA (50 µM) for 72 h. GAPDH and β-Actin were used as loading controls. Bars underneath the blots show quantification for ATGL and pAKT protein expression normalized to respective loading controls. **c** Representative BODIPY™ immunofluorescence staining images of LNCaP cells under different treatment conditions: DMSO (vehicle control), NG-497 (2,2 µM), 2-PMPA (50 µM), and NG-497 (2,2 µM) + 2-PMPA (50 µM) for 72 h. BODIPY™ staining is indicated by green dots. Cells were counterstained with DAPI to visualize nuclei.

Having confirmed an inhibitory effect of NG-497 on ATGL activity, we assessed its impact on cell proliferation across our panel of cell lines (Fig. 5a). LNCaP cells displayed marked sensitivity to NG-497, with a low IC_50_ value of 2.2 µM, indicating high vulnerability to ATGL inhibition. In contrast, PC3, 22RV1, and DU145 cells exhibited significantly greater resistance, with IC_50_ concentrations of 51 µM, 27 µM, and >80 µM (not reached within the tested concentration range), respectively. To elucidate potential mechanisms underlying the differential sensitivity to ATGL inhibition, we assessed the impact of NG-497 and the PSMA inhibitor 2-(Phosphonomethyl)pentanedioic acid (2-PMPA) on the expression of ATGL, PSMA, and PSMA-dependent PI3K-Akt signalling (Fig. 5b). Following treatment with NG-497 at IC_50_ concentrations, western blot analysis revealed consistent upregulation of ATGL in PC3, 22RV1 and DU145 cells, suggesting a compensatory response to ATGL inhibition. LNCaP cells, which expressed high levels of ATGL at baseline, maintained stable ATGL expression across all conditions. Treatment with the PSMA inhibitor 2-PMPA had no effect on ATGL expression, neither as a single agent nor in combination with NG-497. Moreover, PSMA protein expression remained unaffected by single and combined treatments in PSMA-positive LNCaP and 22RV1 cells, while no PSMA expression was detected in PSMA-negative PC3 and DU145 cells within the different conditions. AKT phosphorylation appeared to be slightly reduced upon single NG-497 treatment and in combination with 2-PMPA in PTEN-negative LNCaP and PC3 cells. In PTEN-positive cells, pAKT remained undetectable in all conditions.

To directly assess the functional consequences of ATGL inhibition on intracellular lipid storage, we performed BODIPY™ staining to visualise lipid droplet accumulation in treated cells (Fig. 5c, and Supplementary Fig. S5). NG-497 treatment led to a marked accumulation of intracellular lipid droplets in LNCaP, PC3, and DU145 cells, but notably not in 22RV1 cells, most probably due to high HSL levels in these cells (Supplementary Fig. S4b), which might compensate for ATGL inhibition. Combination treatment with NG-497 and 2-PMPA also resulted in lipid droplet accumulation to a similar extent as NG-497 treatment alone, suggesting that the observed lipid accumulation in the combination treatment is primarily driven by ATGL inhibition, and that 2-PMPA does not significantly alter this NG-497-induced phenotype.

To genetically validate the differential pharmacological ATGL inhibitor sensitivity, we performed proliferation assays following siRNA-mediated knockdown of ATGL and PSMA in LNCaP and 22RV1 cells cultivated in HPLM (Supplementary Fig. S6a, b). Consistent with the pharmacological data, ATGL knockdown significantly reduced LNCaP proliferation (*P*_*adj*_ = 0.012) while 22RV1 cells showed a reduction in proliferation but no statistically significant impairment (adjusted *P*_*adj*_ = 0.075), suggesting that the differential drug sensitivity reflects a genuine difference in ATGL dependency. PSMA knockdown showed less strong effects and did not significantly alter proliferation in either cell line.

ATGL and PSMA knockdown efficiency in LNCaP and 22RV1 cells was confirmed by western blot analysis (Supplementary Fig. S6c). ATGL knockdown upregulated PSMA 2.4-fold in LNCaP but reduced it in 22RV1, while PSMA knockdown did not alter ATGL in LNCaP but reduced it in 22RV1, revealing cell-line-specific cross-regulation. Notably, both knockdowns reduced full-length AR protein levels in both cell lines, including AR-V7 in 22RV1, which was paralleled by compensatory pAKT upregulation in PTEN-negative LNCaP cells. In order to study a potential androgen receptor (AR) dependency of ATGL and PSMA, we exposed LNCaP and 22RV1 cells to IC25 and IC50 doses of enzalutamide, which resulted in decreased AR expression in both cell lines and induction of pAKT in LNCaP cells (Supplementary Fig. S6d). Moreover, enzalutamide treatment increased both ATGL and PSMA levels in both cell lines treated, suggesting a reciprocal regulatory network in which AR signalling modulates PSMA and ATGL expression while disruption of either metabolic protein attenuates AR levels.

**Fig. 6:**
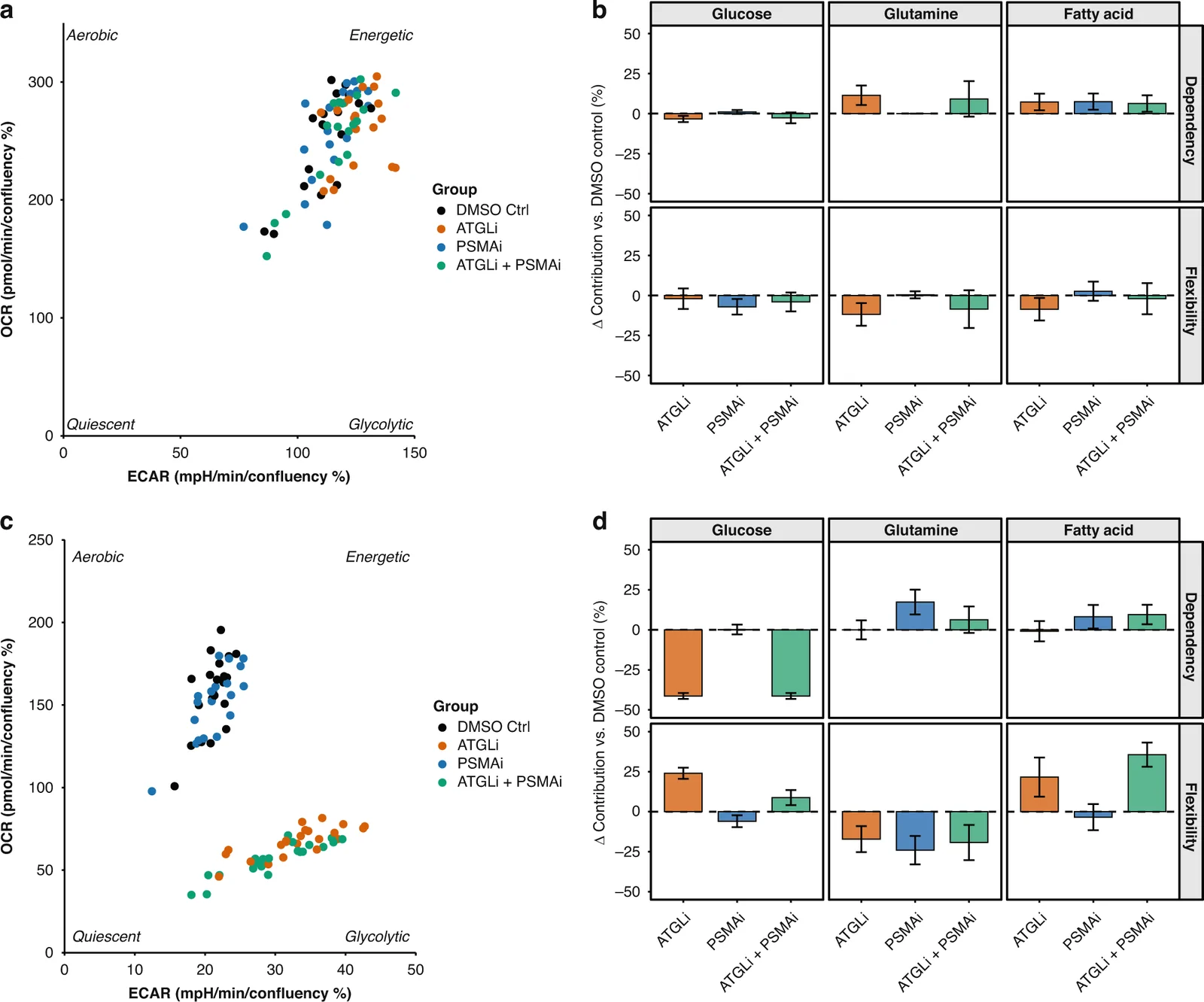
Seahorse XF Mito Fuel Flex Test profiling under pharmacological inhibition of ATGL and PSMA in LNCaP and 22RV1 cells. Energy maps (**a**, **c**) display OCR plotted against ECAR for each individual well at the third baseline measurement before pathway inhibitor injection. Each dot represents one well irrespective of its subsequent measurement condition. Delta contribution plots (**b**, **d**) present the mean change in dependency (upper) and flexibility (lower) for glucose, glutamine, and fatty acid oxidation relative to the vehicle control (DMSO). Error bars represent the propagated standard error of the mean. These assays were performed as single biological experiments with 2 to 4 technical replicate wells per measurement condition after quality control. **a** Energy map for LNCaP cells treated with ATGLi (NG-497), PSMAi (2-PMPA), or the combination. **b** Delta contribution plots for LNCaP inhibitor-treated cells relative to DMSO control. **c** Energy map for 22RV1 cells treated with ATGLi, PSMAi, or the combination. **d** Delta contribution plots for 22RV1 inhibitor-treated cells relative to DMSO control.

Following up on the distinct metabolic profiles described above, we performed Seahorse XF Mito Fuel Flex Test assays under pharmacological inhibition and genetic silencing of ATGL and PSMA in LNCaP and 22RV1 cells to assess their metabolic profiles under perturbation. Under pharmacological ATGL inhibition, LNCaP cells showed only modest shifts in fuel utilisation and failed to expand their metabolic flexibility, consistent with intrinsic metabolic rigidity (Fig. 6a, b). In contrast, 22RV1 cells showed a strong shift towards higher anaerobic glycolysis following ATGL inhibitor treatment at baseline (Fig. 6c, d). When challenged with additional fuel pathway blockade, 22Rv1 cells retained and expanded their reserve capacity to resume mitochondrial glucose and fatty acid oxidation. Genetic silencing of ATGL recapitulated the rigid LNCaP response to the ATGL inhibitor but not the dramatic 22RV1 rewiring, where cells instead reached a compensated steady state, consistent with the lower effects observed in the proliferation assay (Supplementary Fig. S7a–d). PSMA perturbation increased glutamine dependency in both cell lines, with striking quantitative convergence between pharmacological and genetic approaches in 22RV1, while chronic PSMA knockdown additionally drove 22RV1 cells toward broadly elevated dependency and contracted flexibility across all fuel axes.

In conclusion, these data indicate that LNCaP cells are highly dependent on ATGL-mediated lipolysis for proliferation, a vulnerability that can be traced back to their intrinsic metabolic rigidity.

## Discussion

In this study, a combination of in silico, proteomic, and epigenetic analyses of prostate cancer specimens revealed a strong correlation between PSMA and ATGL expression and identified ATGL-driven lipolysis as a metabolic vulnerability in a subset of prostate cancer. Both pharmacological inhibition and genetic silencing of ATGL most potently impaired the viability of LNCaP cells, which exhibit a fatty acid-dependent and metabolically inflexible phenotype, compared to the other prostate cancer cell lines tested. These findings support the therapeutic potential of targeting ATGL in metabolically inflexible prostate cancer and suggest that metabolic profiling may serve as a stratification tool to identify patients likely to benefit from ATGL-directed intervention.

In contrast to the strong PSMA-ATGL correlation observed in our validation cohort, western blot analysis did not demonstrate a consistent correlation pattern between PSMA and ATGL expression in prostate cancer cell lines, which may reflect culture-induced metabolic and epigenetic adaptation [25, 26] as well as variable AR expression [27]. However, this observation is consistent with our central finding that PSMA and ATGL expression levels alone do not predict sensitivity to ATGL inhibition, and that the overall metabolic network architecture is the critical determinant.

Metabolic rewiring towards fatty acid metabolism is recognised as an early hallmark of prostate cancer progression, driven by lifting the zinc-mediated block of citrate cycle aconitase, thereby restoring citrate oxidation and enhancing fatty acid oxidation [14, 28–30]. Therefore, we propose that ATGL-driven lipolysis may play a significant, yet-to-be-defined, role in early prostate cancer metabolic adaptation, potentially acting as an important metabolic facilitator rather than a direct oncogenic driver per se in this context. The observed age-related increase in PSMA and ATGL expression aligns with the emerging paradigm of age-induced metabolic reprogramming in cancer progression [31]. Further research is warranted to fully elucidate the mechanistic interplay and the possibility of a coordinated regulatory mechanism between PSMA and ATGL in prostate cancer initiation and progression.

Using NG-497, we confirmed its potent inhibitory effect on ATGL activity, observing a reduction in triglyceride hydrolysis and a concomitant accumulation of intracellular lipid droplets. The magnitude of triglyceride hydrolase activity inhibition and subsequent lipid droplet accumulation observed in our cell lines is consistent with previous reports in cancer cells using NG-497 and the murine-specific ATGL inhibitor Atglistatin [19, 20]. Interestingly, 22RV1 cells exhibited only a minimal increase in lipid droplet accumulation upon NG-497 treatment. We attribute this relative resistance to lipid accumulation to the constitutively high expression of HSL in 22RV1 cells compared to the other tested cell lines. HSL and ATGL function sequentially in the lipolytic cascade, with ATGL catalysing the initial rate-limiting step of triglyceride hydrolysis to diglyceride, followed by HSL-mediated conversion of diglyceride to monoglyceride [15]. Although HSL exhibits lower triglyceride hydrolase activity than ATGL, elevated HSL expression may provide sufficient lipolytic capacity to compensate for ATGL inhibition, thereby maintaining lipolytic flux and limiting lipid droplet accumulation even in the presence of NG-497. Notably, LNCaP cells displayed the greatest reduction in cell viability following NG-497 treatment. However, the relative resistance of 22RV1 cells despite comparable PSMA expression indicates that the therapeutic window for ATGL inhibition is defined not solely by target expression but by the metabolic flexibility of the tumour.

Our metabolic profiling revealed that despite shared fatty acid dependency, LNCaP and 22RV1 cells differ fundamentally in their capacity for metabolic adaptation. This divergence proved functionally consequential under perturbation, as LNCaP cells failed to expand their metabolic flexibility upon ATGL inhibition while 22RV1 cells executed coordinated metabolic rewiring. Notably, the rewiring observed under acute pharmacological inhibition in 22RV1 was absent following gradual genetic silencing, where cells instead reached a compensated steady state. This discordance is consistent with the framework proposed by Weiss et al. [32], in which acute pharmacological shock captures the immediate stress response before compensatory pathways can be mobilised. From a therapeutic perspective, the acute response is arguably more relevant because clinical drug exposure produces repeated pharmacological shocks. Awad et al. [20] independently demonstrated that ATGL inhibition induces a compensatory glycolytic shift in C4-2 cells and that co-targeting ATGL and glycolysis produces synergistic cell killing, further underscoring that metabolic escape routes are a central consideration when targeting ATGL. This framework of metabolic rigidity versus plasticity may also help reconcile the broader controversy.

ATGL’s role across cancer types, with studies reporting both oncogenic [20, 21, 33–35] and tumour-suppressive [16–18] functions. We propose that the outcome of ATGL inhibition is not determined by ATGL expression itself but by the metabolic flexibility of the tumour. Cells that cannot compensate are vulnerable, while metabolically plastic cells survive by redirecting flux through alternative pathways, or may even benefit from reduced lipolytic flux through mitigated lipotoxicity [36].

Several molecular features likely underlie the differential metabolic plasticity of these cell lines. First, 22RV1 cells express high levels of c-MYC, a driver of mitochondrial glutaminolysis that provides a stable energetic backbone during ATGL blockade. Second, high HSL expression in 22RV1 cells provides compensatory lipolytic capacity when ATGL is blocked. Third, the PTEN-null status of LNCaP cells drives constitutive PI3K/AKT/mTORC1 activation, which suppresses catabolic rescue programs such as autophagy and lipophagy [37], potentially limiting their capacity to mobilise intracellular lipid stores through alternative routes. 22RV1 cells retain wild-type PTEN and therefore regulated mTORC1 signalling. Furthermore, AMPK activation under metabolic stress has been reported to be more robust in 22RV1 than in LNCaP cells [38], providing an additional molecular basis for their metabolic plasticity. PSMA perturbation further distinguished the cell lines, with pharmacological and genetic approaches converging quantitatively on the glutamine axis in 22RV1 but diverging for other fuel parameters, highlighting the importance of perturbation mode when interpreting metabolic profiling data. Notably, chronic PSMA knockdown eroded the inherent metabolic plasticity of 22RV1 cells, suggesting that sustained PSMA-directed strategies may sensitise otherwise resistant tumours. In addition to the enrichment of lipid metabolism pathways, GSEA of our proteomics data revealed a significant downregulation of PI3K-Akt signalling and concurrent upregulation of PTEN regulation in PSMA-high prostate tumours, suggesting that PSMA-dependent PI3K-Akt signalling is counteracted by PTEN in PTEN-proficient tumours. Given PTEN’s established role as a critical negative regulator of the PI3K-Akt pathway and a major tumour suppressor in prostate cancer [39], these findings suggest a potentially paradoxical interplay between PSMA expression and PI3K-Akt signalling in our patient cohort. While previous studies have reported a positive correlation between PSMA expression and PI3K-Akt activation, with PSMA inhibition leading to PI3K-Akt suppression and tumour regression [10], our proteomics data suggest an inverse relationship in the context of high endogenous PSMA expression in patient tumours. Consistent with this, our western blot analysis revealed that while high PSMA expression in PTEN-deficient LNCaP cells was associated with positive pAKT expression, potentially reflecting unopposed PI3K-Akt signalling in the absence of PTEN, this effect was not observed in PTEN-wild-type 22RV1 cells. Conversely, PC3 cells demonstrated similarly high levels of pAKT as LNCaP cells despite lacking PSMA. This observation may reconcile seemingly disparate findings in the literature, as Kaittanis et al. [10] reported increased pAKT activation in 22RV1 cells specifically upon exposure to high concentrations of PSMA substrate, an experimental context distinct from the elevated endogenous PSMA protein expression we observed in patient tumours and cell lines. Our data thus suggest that while supraphysiological levels of PSMA substrate may indeed promote PI3K-Akt activation even in PTEN-wild-type cells, endogenous PSMA expression, particularly in the presence of functional PTEN, may not necessarily translate to a net increase in PI3K-Akt signalling in vivo. While pAKT levels appeared slightly reduced following NG-497 treatment in PTEN-deficient cells, PSMA knockdown in LNCaP produced a pronounced increase in pAKT, indicating that the relationship between the PSMA/ATGL axis and PI3K-AKT signalling is complex and perturbation-dependent rather than a simple feedback mechanism.

Our data reveal that AR blockade by enzalutamide dose-dependently upregulated ATGL protein in both LNCaP and 22RV1 cells, a previously unreported finding that adds a new dimension to the known effects of AR-targeted therapies on prostate cancer metabolism. PSMA was similarly upregulated in 22RV1, consistent with the established inverse relationship between AR signalling and PSMA expression [40, 41]. Conversely, siRNA-mediated knockdown of either ATGL or PSMA reduced AR in both cell lines, consistent with published findings for PSMA depletion [42] and suggesting a reciprocal regulatory network between AR signalling and the PSMA/ATGL axis. This bidirectional cross-regulation has potential clinical implications, as enzalutamide, a standard of care in advanced prostate cancer, may inadvertently upregulate both therapeutic targets, potentially priming tumours for subsequent ATGL-directed intervention. This rationale is consistent with the finding that fatty acid oxidation sustains prostate cancer cells under anti-androgen therapy and that inhibiting lipid catabolism sensitises them to it [43].

In conclusion, our findings establish ATGL-driven lipolysis as a metabolic vulnerability in prostate cancer that is determined by intrinsic metabolic rigidity, a principle that likely translates to tumours in vivo. While preclinical safety data for NG-497 are needed, the established tolerability and efficacy of the related murine-specific ATGL inhibitor Atglistatin in animal models [19, 20, 44] provides a rationale for pursuing in vivo efficacy and safety studies with NG-497. Metabolic profiling in combination with ATGL expression analysis will be essential to identify patients most likely to benefit from ATGL inhibition, offering a framework for precision metabolic therapy in prostate cancer.

## Material and methods

### In silico TCGA-PRAD data analysis

Publicly available data from The Cancer Genome Atlas Prostate Adenocarcinoma project (TCGA-PRAD, *n* = 497 primary solid tumours) [24] were downloaded via the R package RTCGAToolbox [45]. Pre-normalised RSEM (RNA-Seq by Expectation Maximisation) TPM (Transcripts Per Million) gene expression values (RNASeq2GeneNorm dataset) were utilised. Patients were stratified into *FOLH1*-high (top 10% expression) and* FOLH1*-low (bottom 10%) groups based on *FOLH1* TPM values. Differential gene expression analysis between these groups was performed using the limma R package [46], defining significance as an adjusted *P* < 0.05 and an absolute Log2FC > 1. Functional enrichment analysis for KEGG pathways on the resulting differentially expressed genes was conducted using the TCGAbiolinks R package [47]. DNA methylation data (Illumina Human Methylation 450k platform beta values) for the same 497 tumours were downloaded using TCGAbiolinks. Differentially methylated CpG sites between the *FOLH1*-high and -low expression groups (defined by RNA-seq) were identified using functionalities within TCGAbiolinks, calculating the difference in mean beta values between groups and applying a Wilcoxon test with Benjamini–Hochberg correction. Significantly differentially methylated CpG sites were defined as having an absolute difference in mean beta values > 0.1 and an *P*_*adj*_ < 0.01.

### Shotgun proteomics & RRBS

Patients undergoing radical prostatectomy were included based on pre-operative [^68^Ga]Ga-PSMA PET/MRI demonstrating primary tumour heterogeneity (PSMA-high and -low regions) and PET-positive regional lymph node metastasis. Archived FFPE blocks corresponding to heterogeneous primary tumour regions were selected using imaging data. PSMA-high and -low areas within sections from these blocks were confirmed by PSMA IHC. PSMA-high regions were defined by strong immunohistochemical staining intensity (semiquantitative score +++) and PSMA-low regions by weak staining intensity (score +), reflecting the intratumoural heterogeneity of PSMA expression in prostate cancer tissue, which is rarely completely PSMA-negative. FFPE sections were then macrodissected to isolate approximately 1 mm³ of tumour tissue from immunohistochemically confirmed PSMA-high and -low regions for parallel processing. For proteomics, macrodissected tissue underwent standard deparaffinization (xylene) and rehydration (graded ethanol series) before short-term storage at −80 °C. Protein extraction and digestion using an adapted FASP protocol [48] were performed by the Max Perutz Labs Mass Spectrometry facility (Vienna, Austria; see Supplementary Methods). For RRBS, DNA was extracted automated with the Qiagen EZ1 using the Qiagen EZ1 DNA FFPE Kit following the manufacturer's instructions for sample preparation.

Protein raw intensity data were filtered in Perseus (v1.6.10.43) [49] to remove contaminants, “only identified by site” proteins, and proteins not detected in >60% of samples in at least one comparison group. Differential expression analysis was performed using a custom R script with the DEP package [50]. Following confirmation of left-censored missing values (Supplementary Methods Fig. 1), data were processed using variance stabilizing normalisation (vsn) and group-based MinProb imputation (*q* = 0.01). GSEA was conducted using the clusterProfiler package [51] with KEGG and REACTOME pathway databases.

RRBS library preparation, high-coverage sequencing and data processing were performed by the Biomedical Sequencing Facility (CeMM Research Center for Molecular Medicine of the Austrian Academy of Sciences, Vienna, Austria; see Supplementary Methods). Processed methylation data (Biseq Bed files) were analysed using custom R scripts with bsseq [52] and DSS [53] packages. Following DML testing, DMRs were identified requiring a minimum region length of 50 bp containing at least 3 CpGs, with at least 80% of CpGs within the region meeting a significance threshold of *P*_adj_ < 0.05. DMR-associated genes were subjected to overrepresentation analysis using clusterProfiler against KEGG and REACTOME gene sets.

### TMA construction & Immunohistochemistry

A validation cohort was established using archived FFPE tissue blocks from treatment-naïve patients who underwent radical prostatectomy at the AKH Wien (Vienna General Hospital, Vienna, Austria) between 2016 and 2021, with pathologically confirmed prostate adenocarcinoma Gleason Score 3+4, 4+3 or 4+4 (patient details in Supplementary Table 2). Representative tumour areas were marked on diagnostic H&E slides. Duplicate 2 mm cores were extracted from selected tumour regions of each patient block and assembled into TMAs using a Galileo TMA CK3500 system. TMAs were sectioned at 2 µm thickness. IHC staining was performed using automated platforms (Ventana Autostainer, Leica Autostainer) for PSMA, Ki-67, PGM1(CD68), AR, Synaptophysin, and c-MYC, while ATGL staining was performed manually using the ImmPRESS® HRP Universal Polymer Kit following the manufacturer’s instructions. Primary antibodies used were: PSMA (Cell Marque, 327R-18, ready-to-use), Ki-67 (Ventana, 790-4286/Clone 30-9, ready-to-use), PGM1(CD68) (Dako, M0876/Clone PG-M1, 1:100), AR (Dako, M3562/Clone AR441, 1:100), Synaptophysin (Ventana, 790-4407/Clone SP11, ready-to-use), c-MYC (Epitomics, AC-0116/Clone EP121, 1:100), and ATGL (Invitrogen, PA5-119928, 1:200). Stained TMA slides were digitized using a 3DHISTECH Pannoramic 250 scanner. Scoring was performed by a pathologist blinded to clinicopathological data. PSMA, ATGL, AR, Synaptophysin, and c-MYC expression were quantified using the H-score method as previously described [54]. PGM1(CD68)-positive tumour-associated macrophages were quantified by averaging cell counts per high-power field across three distinct dense, non-inflamed stromal areas per core. Pairwise correlations between marker expression levels and clinicopathological variables were assessed using Spearman correlation coefficients. Statistical testing was performed on pre-selected biologically motivated associations without adjustment for multiple comparisons.

### Cell culture

The human prostate cancer cell lines LNCaP, PC3, 22RV1, DU145, LAPC4, DUCaP, and VCaP were obtained from the American Type Culture Collection (ATCC). These lines were routinely maintained in RPMI-1640 medium (Gibco, Thermo Fisher #11875-093) supplemented with 10% Fetal Bovine Serum (Gibco, Thermo Fisher # A56707-01) and 1% Penicillin/Streptomycin (Gibco, Thermo Fisher #15140122). For indicated experiments, cells were cultured in HPLM (Gibco, Thermo Fisher #A48991-01). The non-neoplastic prostate epithelial cell line RWPE-1 (ATCC) was cultured using a Keratinocyte Serum-Free Medium (K-SFM) kit (Gibco, Thermo Fisher #17005075), including supplementation with bovine pituitary extract and epidermal growth factor provided within the kit, according to the manufacturer’s instructions. All cell lines were maintained at 37 °C in a humidified atmosphere with 5% CO₂.

### siRNA-mediated knockdown

Transient knockdowns were performed using Lipofectamine^TM^ 2000 Reagent (Thermo Fisher #11668027) with Silencer Select Pre-designed siRNAs (Thermo Fisher #4392420) targeting *PNPLA2* (siRNA ID s32682) or *FOLH1* (siRNA ID s5328) at 30 nM final concentration following the manufacturer’s instructions.

### Western blot analysis

Snap-frozen cell pellets were processed for SDS-PAGE and western blot analysis as previously described [55]. Antibodies and buffers are listed in Supplementary Methods. Western blot band intensities were quantified using Image Lab Software version 6.1.0 (Bio-Rad).

### Enzalutamide treatment

LNCaP and 22RV1 cells were seeded at 300,000 cells per well in 6-well plates in HPLM. After 24 h, cells were treated with enzalutamide (MedChemExpress #HY-70002) at IC25 and IC50 concentrations (LNCaP, 20 µM and 40 µM; 22RV1, 22.5 µM and 45 µM, respectively) or vehicle control (DMSO) for 72 h. IC25 and IC50 values were derived from published data [56, 57]. Protein lysates were harvested for western blot analysis as described above.

### Triglyceride hydrolase activity assay

Frozen cell pellets were sonicated in ice-cold solution A [0.25 M sucrose, 1 mM EDTA, 1 mM dithiothreitol, pH 7.0] supplemented with protease inhibitors (20 µg/mL leupeptin, 2 µg/mL antipain, 1 µg/mL pepstatin; Carl Roth GmbH, Karlsruhe, Germany). Cell lysates were centrifuged for 10 min at 4 °C, and 1000 ×  *g* and the fat-free infranatant was collected. 100 µg of cell lysate protein was incubated with a TG substrate. TG substrate was prepared by emulsifying 0.3 mM, 10 µCi/mL triolein [9,10-3H(N)] (NET431L, Perkin-Elmer, Waltham, MA), and 45 µM PC/PI (3:1; M:M) in 100 mM potassium phosphate buffer (pH: 7.0) by sonication on ice (Virsonic 475, Virtis, Gardiner, NJ). As FA acceptor, 5% FA-free BSA (Sigma-Aldrich) was added. Cell lysates were incubated with TG substrate for 1 h at 37 °C in a water bath. After incubation, the reaction was terminated by the addition of 650 µl methanol/chloroform/heptane (10/9/7; vol/vol/vol) and 200 µL of 0.1 M potassium-carbonate/0.1 M boric acid (pH: 10.5). The samples were mixed and centrifuged for 10 min at 1000 × *g*. An aliquot of 200 µL of the upper aqueous phase was transferred to 2 mL Rotiszint eco plus (Roth). Radioactivity was analysed using liquid scintillation counting (Roth; Tri-Carb 2100TR, Packard Instrument Company). TG hydrolase activity was determined as released FA per hour and mg protein [58].

### Cell proliferation assay

96-well plates (Thermo Scientific, Thermo Fisher #165305) were coated with Poly-D-Lysine (Gibco, Thermo Fisher #A38904-01) according to the manufacturer’s instructions prior to cell seeding. Cells were seeded as technical triplicates in HPLM medium at the following densities: LNCaP, 7000 cells/well; PC3, 3500 cells/well; 22RV1, 8500 cells/well; and DU145, 4000 cells/well. NG-497 (MedChemExpress, #HY-148756), dissolved in DMSO, was added at various concentrations. Treatment started 24 h after seeding. Medium containing fresh NG-497 or vehicle control (DMSO) was replenished every 12 h for a total treatment duration of 72 h. The final DMSO concentration in all wells was normalised to 0.1%. Cell proliferation was assessed after 72 h using the CyQUANT™ NF Cell Proliferation Assay Kit (Invitrogen, Thermo Fisher #C35006) following the manufacturer’s protocol, with fluorescence measured using the Agilent BioTek Synergy H1 microplate reader. Cell viability (%) was calculated relative to vehicle-treated controls (representing 100% viability) and a maximum inhibition control (5 µM Staurosporin; Sigma-Aldrich, #569397, representing 0% viability) using the formula: % Viability = ((Well Value - Average Staurosporin Control) / (Average Vehicle Control - Average Staurosporin Control)) * 100. IC_50_ values were calculated using GraphPad Prism v8.4.3 with a non-linear regression (log[inhibitor] vs. normalised response) model.

For knockdown proliferation assays, cells were seeded in HPLM on Poly-D-Lysine-coated 96-well plates at densities of 4000 (LNCaP) or 5000 (22RV1) cells per well. siRNA transfection was performed 24 h after seeding as described above. Cell proliferation was assessed 72 h post transfection using the CyQUANT™ NF assay as described above. Four independent biological replicates with four technical replicates each were performed per cell line. Statistical significance was assessed using paired *t*-tests against the Lipofectamine vehicle control with Holm correction for multiple comparisons.

### Seahorse XF Mito Fuel Flex Test Assay

Cellular OCR and metabolic dependencies were assessed using the Seahorse XF Mito Fuel Flex Test kit (Agilent Technologies, #103260-100) on a Seahorse XFe96 Analyser (Agilent Technologies). Cells were seeded into Seahorse XFe96 Cell Culture microplates (Agilent Technologies, #103794-100) 24 h prior to the assay in HPLM medium at densities of 20,000 (LNCaP), 18,000 (PC3), 30,000 (22RV1), or 30,000 (DU145) cells per well, with 3–4 technical replicates per condition. Immediately before the assay, the culture medium was replaced with Seahorse XF DMEM assay medium kit (Agilent Technologies, #103680-100), supplemented with Pyruvate (0.05 mM), Glutamine (0.55 mM), and Glucose (5 mM) included with the kit to reflect HPLM concentrations. The assay, including inhibitor injections (Etomoxir, BPTES, UK5099) and OCR measurements, as well as subsequent data analysis to determine fuel dependency and flexibility, were performed as previously described [59].

For pharmacological perturbation experiments, cells were seeded in HPLM and treated overnight with NG-497 at predetermined IC_50_ concentrations or 50 µM 2-PMPA, alone or in combination. Inhibitors were re-added when changing to Seahorse assay medium approximately 1 h before the assay readout. For knockdown perturbation experiments, cells were transfected in 6-well plates as described above and reseeded onto Seahorse microplates 48 h post transfection at densities of 20,000 (LNCaP) or 30,000 (22RV1) cells per well. The assay was performed 24 h after reseeding using the same protocol and analysis pipeline as above.

### Lipid droplet staining (BODIPY™)

Cells were seeded onto Poly-D-Lysine-coated 8-well chamber slides (ibidi #80806) at densities of 50,000 cells/well (LNCaP, DU145) or 30,000 cells/well (PC3, 22RV1). Treatments with NG-497 (calculated IC_50_ concentrations), 50 µM 2-PMPA (MedChemExpress, #HY-100788), or vehicle control (0.1% DMSO) were conducted in HPLM for 72 h, with media and treatment being refreshed every 12 h, mirroring the conditions used in the proliferation assays. After 72 h, cells were washed once with PBS and incubated with 2 µM BODIPY™ 493/503 FL dye (Invitrogen, Thermo Fisher #D3922) in PBS for 15 min at 37 °C. Following two further PBS washes, cells were fixed with 4% paraformaldehyde in PBS for 30 min at room temperature. After fixation and three 5-min washes in PBS, nuclei were counterstained with DAPI (1 µg/mL) for 20 min. Stained cells were imaged using a Zeiss LSM 700 confocal microscope controlled by ZEN software version 3.9. Representative images were acquired using either a 16× or 40× objective. Notably, images for DU145 cells were acquired using the 40× objective, while images for LNCaP, PC3, and 22RV1 were acquired using the 16× objective. For consistent visual representation in the figures, the 16× images were digitally magnified using ImageJ Fiji version 1.54p [60] to approximate the scale of the 40× images.

### Use of large language models (LLM)

Assistance from the large language models Gemini 2.5 Pro Preview (Google) and Claude Opus 4.6 (Anthropic) was utilised for language editing and refinement of the manuscript text. User-provided drafts were iteratively improved based on suggestions from the LLM regarding clarity, conciseness, grammar, and overall flow. All final wording and scientific content were determined by the authors, who take full responsibility for the manuscript.

### Ethics declaration

This study involving human tissue samples and associated clinical data adhered to the Good Scientific Practice guidelines of the Medical University of Vienna and the “Declaration of Helsinki”. The study was reviewed and approved by the ethics commission of the Medical University of Vienna (approval number: 1903/2018). Given the retrospective nature of the study and the exclusive use of archived tissue samples, the ethics commission waived the requirement for informed consent.

## Supplementary information

Legends for supplemental material

Supplementary Figure S1

Supplementary Figure S2

Supplementary Figure S3

Supplementary Figure S4

Supplementary Figure S5

Supplementary Figure S6

Supplementary Figure S7

Supplementary Table 1

Supplementary Table 2

Supplementary Dataset 1

Supplementary Dataset 2

Supplementary Methods

Reproducibility checklist

## Data Availability

The mass spectrometry proteomics data have been deposited to the ProteomeXchange Consortium via the PRIDE [61] partner repository with the data set identifier PXD065989. The RRBS dataset has been deposited in the NCBI Gene Expression Omnibus under accession number GSE303504.
