# Supplementary material for: Adipose triglyceride lipase driven lipolysis as a targetable metabolic vulnerability in prostate cancer with intrinsic metabolic inflexibility
Source: Br J Cancer. 2026 Jul 23;135(8):1342–55. doi: 10.1038/s41416-026-03545-4 (PMC13642917; doi:10.1038/s41416-026-03545-4)
Supplement: Supplementary file 2 [file 41416_2026_3545_MOESM13_ESM.docx]

**Supplementary Methods**

*LC-MS/MS shotgun proteomics*

*Sample preparation for mass spectrometry analysis*

The samples were prepared using an FFPE-FASP protocol adapted from Wiśniewski JR et al. [1]. Deparaffinized samples were lysed in 60 μL lysis buffer consisting of 0.3 M Tris-HCl, pH 8.5, 0.1 M DTT, and 2% SDS at 95°C in a heating block with agitation (600 rpm) for 1 h. The crude extracts were clarified by centrifugation at 16000 x g at 20°C for 10 min and the supernatants were transferred into new tubes. 540 μL urea buffer consisting of 8M urea in 0.1 M Tris-HCl, pH 8.5 was added to the tubes to dilute the SDS. Microcon-30 filter units with 30 kDa cutoff were washed with 200 μL urea buffer at 12000 x g at 20°C for 15 min. 300 μL diluted sample were applied onto the filter and centrifuged at 12000 x g at 20°C for 20 min. This step was repeated with the remaining 300 µL to load all sample on the filter. The filter was washed with 200 μL urea buffer and centrifuged at 12000 x g at 20°C for 20 min. 100 μL 0.05 M iodoacetamide in urea buffer was added to the filter and mixed slowly on a shaker for 1 min following incubation without mixing for 30 min in the dark and subsequent centrifugation at 12 000 g at 20°C for 20 min. The filters were washed three times with 100 μL urea buffer at 12000 x g at 20°C for 15 min, and then three times with 100 μL 0.1 M Tris-HCl pH 8.5 at 12000 x g at 20°C for 10 min. The filter units were transferred to new collection tubes and 20 μL 0.1M Tris-HCl pH 8.5 buffer was added to the bottom of the new collection tubes to prevent the filter from drying out during digestion. 1 μg trypsin in 40 μL 0.1M Tris-HCl pH 8.5 buffer (enzyme to protein ratio about 1:50) were added onto the filters. The tubes were sealed with Parafilm and incubated at 37°C overnight (18h). Next day, the filter tubes were centrifuged at 12000 x g at 20°C for 20 min to elute the peptides. 40 μL 0.1M Tris-HCl pH 8.5 buffer was added onto the filter and centrifuged at 12000 x g at 20°C for 10 min. After addition of 5 μL 10% TFA the peptides were desalted using C18 Stagetips [2].

*Liquid chromatography separation coupled to mass spectrometry*

Peptides were separated on an Ultimate 3000 RSLC nano-flow chromatography system (Thermo-Fisher), using a pre-column for sample loading (Acclaim PepMap C18, 2 cm × 0.1 mm, 5 μm, Thermo-Fisher), and a C18 analytical column (Acclaim PepMap C18, 50 cm × 0.75 mm, 2 μm, Thermo-Fisher), applying a segmented linear gradient from 2% to 35% and finally 80% solvent B (80% acetonitrile, 0.1% formic acid; solvent A 0.1% formic acid) at a flow rate of 230 nL/min over 120 min. Eluting peptides were analyzed on a Q Exactive HF-X Orbitrap mass spectrometer (Thermo Fisher), which was coupled to the column with a customized nano-spray EASY-Spray ion-source (Thermo-Fisher) using coated silica emitter tips (New Objective).

*Mass spectrometry data acquisition and analysis*

The mass spectrometer was operated in data-dependent acquisition mode (DDA), survey scans were obtained in a mass range of 375-1500 m/z with lock mass activated, at a resolution of 120k at 200 m/z and an AGC target value of 3E6. The 20 most intense ions were selected with an isolation width of 1.2 m/z, fragmented in the HCD cell at 28% collision energy and the spectra recorded for max. 80 ms at a target value of 1E5 and a resolution of 30k. Peptides with a charge of +1, >+7, or unassigned charge state were excluded from fragmentation, the peptide match feature was set to preferred, the exclude isotope feature was enabled, and selected precursors were dynamically excluded from repeated sampling for 40 seconds.

Raw data were processed using the MaxQuant software package (version 1.6.0.16, [3]) and the Uniprot human reference proteome (January 2019, www.uniprot.org), target sequences, as well as a database of most common contaminants. The search was performed with full trypsin specificity and a maximum of two missed cleavages at a protein and peptide spectrum match false discovery rate of 1%. Carbamidomethylation of cysteine residues was set as fixed, oxidation of methionine and N-terminal acetylation as variable modifications. For label-free quantification the “match between runs” feature and the LFQ function were activated - all other parameters were left at default.

Figure 1: Proteins with missing values have on average low raw intensities.

*Reduced Representation Bisulfite Sequencing (RRBS)*

*Library preparation*

Reduced Representation Bisulphite Sequencing (RRBS) libraries were generated as described previously [4]. Briefly, up to 100 ng of genomic DNA were digested for 12 hours at 37 °C with 20 U of MspI (R0106L, New England Biolabs, Inc., Ipswich, MA, USA) in 30 μl of 1 x NEB buffer 2. To retain even the smallest fragments and to minimize the loss of material, end preparation and adaptor ligation were performed in a single-tube setup. End fill-in and A-tailing were performed by addition of Klenow Fragment 3’ > 5’ exo-mix (P7010-LC-L, Enzymatics) and dNTP mix (10 mM dATP, 1 mM dCTP, 1 mM dGTP, N0446S, New England Biolabs). After ligation to methylated Illumina TruSeq LT v2 adaptors using T4 DNA Ligase rapid (L6030-HC-L, Enzymatics), the libraries were size selected via a 0.75 x clean-up with AMPure XP beads (A63881, Beckman Coulter). The libraries were pooled based on qPCR data and subjected to bisulfite conversion using the EZ DNA Methylation Direct Kit (D5020, Zymo Research) with changes to the manufacturer’s protocol: conversion reagent was used at 0.9 x concentration, incubation performed for 20 cycles of 1 min at 95 °C, 10 min at 60 °C and the desulfonation time was extended to 30 min. These changes increase the number of CpG dinucleotides covered, by reducing double strand break formation in larger library fragments. Bisulfite-converted libraries were enriched via KAPA HiFi HS Uracil+ RM (07959079001, Roche Molecular Systems, Inc.). The minimum number of enrichment cycles was estimated based on a qPCR experiment. After a 1 x AMPure XP clean-up, library concentrations were quantified with the Qubit Fluorometric Quantitation system (Q32854, Thermo Fisher Scientific, Waltham, MA, USA) and the size distribution was assessed using the High Sensitivity DNA Kit (5067-4626, Agilent, Santa Clara, CA, USA) on a 2100 Bioanalyzer (Agilent) instrument. Sample-specific RRBS libraries were diluted and pooled in equimolar amounts.

*Next-generation sequencing and raw data acquisition*

RRBS pools were sequenced on a HiSeq 4000 instrument (Illumina, San Diego, CA, USA) following a 50-base-pair, single-end recipe. Raw data acquisition (HiSeq Control Software, HCS, HD 3.4.0.38) and base calling (Real-Time Analysis Software, RTA, 2.7.7) was performed on-instrument, while the subsequent raw data processing off the instruments involved two custom programs (https://github.com/epigen/picard/) based on Picard tools (2.19.2; https://broadinstitute.github.io/picard/). In a first step, base calls were converted into lane-specific, multiplexed, unaligned BAM files suitable for long-term archival (IlluminaBasecallsToMultiplexSam, 2.19.2-CeMM), thereby annotating an eventual start of NGS adapter sequences in XT tags. In a second step, archive BAM files were demultiplexed into sample-specific, unaligned BAM files (IlluminaSamDemux, 2.19.2-CeMM).

*Data Analysis*

Custom scripts were used to calculate the digestion metrics for the TaqI and MspI restriction enzymes. Unaligned BAM files were converted into FASTQ files and pre-processed with Trimmomatic [5] using options ILLUMINACLIP:RRBS_adapters.fa:2:40:7 SLIDINGWINDOW:4:15 MAXINFO:20:0.50 MINLEN:18. Trimmed NGS reads were aligned to the reference genome with the methylation-aware BSMAP aligner in RRBS mode for MspI (-D C-CGG) and TaqI (-D T-CGA) restriction sites, a filter of 5 Ns (-f 5), a quality threshold of 0 (-q 0), a seed size of 12 (-s 12), a mismatch rate of 0.08 (-v 0.08, or 4 mismatches in 50), a minimal inset size of 20 (-m 20), a maximal insert size of 500 (-x 500) mapping to forward strands only (-n 0). (Options: -D C-CGG -D T-CGA -f 5 -m 20 -n 0 -p 4 -q 0 -r 1 -s 12 -S 0 -u -v 0.08 -V 2 -w 100 -x 500). Methylated bases were called with the biseqMethCalling script (https://github.com/epigen/biseqMethCalling) and the resulting UCSC Genome Browser BED files were converted into BigBed files for integration into a UCSC Genome Browser Track Hub for visual inspection. Internal standards comprised of fully methylated and unmethylated double stranded synthetic oligo sequences were added to each sample to determine the rates of conversion and over-conversion, respectively. The internal standards were aligned with Bismark [6] and methylation calls were evaluated with a custom script.

*Cell Culture*

Figure 2: Concentration differences of important metabolites between RPMI-1640 and HPLM cell culture media.

*Western blot analysis*

*Hunt cell lysis buffer:*

20 mM Tris pH 8, 100 mM NaCl, 1 mM EDTA, 0.5% NP-40, protease inhibitor – Roche

*Milk blocking solution:*

5% skim milk powder, 1% PVP, 0.01% Sodium azide in TBS-T (=TBS containing 1% Triton-X100)

*BSA blocking solution:*

5% BSA, 0.01% Sodium azide in TBS-T (=TBS containing 1% Triton-X100) *BSA blocking solution was used when phosphorylated proteins were detected.

*ECL solutions:*

Amersham ECL Detection Reagents

*Primary antibodies used:*

ATGL CST #2138 - dilution 1:1000, c-MYC CST #18583 - dilution 1:500, HSL proteintech #17333-1-AP - dilution 1:1000, ABHD5 bio-techne #NB110-41576H - dilution 1:1000, p-AKT CST #9271, PSMA CST #12815 - dilution 1:1000, PTEN CST #9559 – dilution 1:1000, ß-ACTIN proteintech #66009-1-Ig – dilution 1:5000, GAPDH .CST #97166.

*Secondary antibodies used:*

Peroxidase AffiniPure™ Rabbit Anti-Mouse IgG, Fcγ fragment specific (JacksonimmunoResearch #315–035-008), Peroxidase AffiniPure™ F(ab′)₂ Fragment Goat Anti-Rabbit IgG, F(ab′)₂ fragment specific (JacksonimmunoResearch #111-036-047)

*References*

[1]	Wiśniewski JR, Ostasiewicz P, Mann M. High Recovery FASP Applied to the Proteomic Analysis of Microdissected Formalin Fixed Paraffin Embedded Cancer Tissues Retrieves Known Colon Cancer Markers. J Proteome Res 2011;10:3040–9. https://doi.org/10.1021/pr200019m.

[2]	Rappsilber J, Mann M, Ishihama Y. Protocol for micro-purification, enrichment, pre-fractionation and storage of peptides for proteomics using StageTips. Nat Protoc 2007;2:1896–906. https://doi.org/10.1038/nprot.2007.261.

[3]	Tyanova S, Temu T, Cox J. The MaxQuant computational platform for mass spectrometry-based shotgun proteomics. Nat Protoc 2016;11:2301–19. https://doi.org/10.1038/nprot.2016.136.

[4]	Klughammer J, Datlinger P, Printz D, Sheffield NC, Farlik M, Hadler J, et al. Differential DNA Methylation Analysis without a Reference Genome. Cell Rep 2015;13:2621–33. https://doi.org/10.1016/j.celrep.2015.11.024.

[5]	Bolger AM, Lohse M, Usadel B. Trimmomatic: a flexible trimmer for Illumina sequence data. Bioinformatics 2014;30:2114–20. https://doi.org/10.1093/bioinformatics/btu170.

[6]	Krueger F, Andrews SR. Bismark: a flexible aligner and methylation caller for Bisulfite-Seq applications. Bioinforma Oxf Engl 2011;27:1571–2. https://doi.org/10.1093/bioinformatics/btr167.
