# Supplementary material for: Adipose triglyceride lipase driven lipolysis as a targetable metabolic vulnerability in prostate cancer with intrinsic metabolic inflexibility
Source: Br J Cancer. 2026 Jul 23;135(8):1342–55. doi: 10.1038/s41416-026-03545-4 (PMC13642917; doi:10.1038/s41416-026-03545-4)
Supplement: Supplementary file 3 [file 41416_2026_3545_MOESM1_ESM.docx]

**Supplementary Figure S1:** Transcriptomic and epigenetic landscape associated with high *FOLH1* expression in prostate cancer (TCGA-PRAD)

**a,** *FOLH1* (PSMA) gene expression levels in TCGA-PRAD prostate tumors (n=497). Samples are stratified into high (top 10%, green, n=55) and low (bottom 10%, orange, n=55) *FOLH1* expression groups. **b,** Hierarchical clustering of top 100 significantly differentially expressed genes in high and low *FOLH1* gene expression groups. Each row corresponds to a single gene, whereas each column corresponds to a single patient sample. Data are presented at Z-score normalized values with red indicating upregulated and blue downregulated genes. **c,** Top enriched canonical pathways associated with differentially expressed genes are visualized according to their *P* value corrected false discovery rate (FDR) as yellow bars. The ratio of genes found in each pathway over the total number of genes in the respective pathway is indicated as a red line. **d,** Mean DNA methylation levels across *FOLH1* gene expression groups as in a) (high, medium, and low) extracted from TCGA-PRAD **e,** Hierarchical clustering of significantly differentially methylated CpG sites (n=661). Each row corresponds to a single CpG site, whereas each column corresponds to a single patient sample.

**Supplementary Figure S2:** Characteristics of proteomic data and top differentially expressed proteins.

**a,** Upset plot showing the number and overlap of protein groups identified in each of the 8 patients included in the patient-matched proteomics analysis. Bars below the graph represent individual patients, and connected black dots indicate the intersection of protein detection across patient groups. Grey dots indicate no overlap with detected proteins. **b,** Comparison of overall DNA methylation levels between PSMA-high (red box) and PSMA-low (blue box) tumour samples.

**Supplementary Figure S3:** PSMA and ATGL protein expression distributions and selected metabolic correlations.

**a,** Violin plot depicting the distribution of PSMA protein expression immunohistochemistry H-scores across the tissue micro-array (TMA) cohort (n=90). **b,** Violin plot depicting the distribution of ATGL protein expression H-scores across the TMA cohort (n=90). **c,** Box plots comparing PSMA protein expression H-scores across Gleason score groups (left) and pathological T stages (T2 and T3) (right). **d,** Box plots comparing ATGL protein expression H-scores across Gleason score groups (left) and pathological T stages (T2 and T3) (right). **e,** Scatter plots and linear regression lines illustrating the correlation between PSMA (left) and ATGL (right) protein expression H-scores and serum triglyceride levels. **f,** Scatter plot and linear regression line illustrating significant positive correlation between ATGL with serum cholesterol levels in the TMA cohort.

**Supplementary Figure S4:** Supporting data for cell line metabolic characterization using human plasma-like medium (HPLM)

**a,** Triglyceride hydrolase activity assay in selected prostate cancer cell lines cultured in HPLM. Cell lysates were incubated with vehicle control (control) or 20 µM NG-497 for 1 hour (NG-497). Released fatty acids (nmol/hour) per mg of protein are shown. Data represent mean ± SEM (n=3). Statistical significance was determined by unpaired t-test (**P* < 0.05, ***P* < 0.01, ****P* < 0.001 vs. vehicle control within each cell line). **b,** Western blot analysis of PSMA, ATGL, HSL, c-MYC and ABHD5 in selected prostate cancer cell lines cultured in RPMI-1640 or HPLM. GAPDH was used as a loading control. **c,** Basal oxygen consumption rate (OCR) in selected prostate cancer cell lines, as measured by Seahorse XF Mito Fuel Flex Test assay. Data represent mean ± SEM (for LNCaP, PC3 and DU145 n=20; for 22RV1 n=23; based on plate-layout and removal of wells for data quality reasons). Statistical significance was determined by One-way ANOVA with Tukey multiple comparison tests including appropriate adjustment for multiple testing (**P* < 0.05, ***P* < 0.01, ****P* < 0.001).

**Supplementary Figure S5:** Additional BODIPY™ Staining Data in PC3, 22RV1, and DU145

**a,** Representative BODIPY™ immunofluorescence staining images of PC3, 22RV1, and DU145 cells under different treatment conditions: DMSO (control), NG-497 (51 µM, 27 µM and 80 µM for PC3, 22RV1 and DU145, respectively), 2-PMPA (50 µM), and NG-497 (51 µM, 27 µM and 80 µM for PC3, 22RV1 and DU145, respectively) + 2-PMPA (50 µM) for 72 hours. BODIPY™ staining is indicated by green dots. Cells were counterstained with DAPI to visualize nuclei.

**Supplementary Figure S6:** Effects of siRNA-mediated knockdown of ATGL and PSMA and enzalutamide treatment on proliferation and protein expression in LNCaP and 22RV1 cells.

**a,** CyQuant® Cell Proliferation Assay in LNCaP cells following siRNA-mediated knockdown of ATGL (ATGL KD) or PSMA (PSMA KD) in HPLM. Bars represent mean fold change relative to Empty Ctrl across four independent biological replicates (four technical replicates each), with individual biological replicates shown as data points. Empty Ctrl, untransfected cells. Lipo Ctrl, lipofectamine vehicle control. Statistical significance was assessed using paired t-tests against Lipo Ctrl with Holm correction for multiple comparisons. * P_adj_ < 0.05, ns not significant. **b**, CyQuant® Cell Proliferation Assay in 22RV1 cells, performed and analyzed as in (a). **c c**, Western blot analysis showing protein expression of PSMA, pAKT, AR, AR-V7, and ATGL in LNCaP and 22RV1 cells 72 h after siRNA-mediated knockdown of ATGL or PSMA. Empty Ctrl refers to untransfected cells. pAKT was not detected in 22RV1 cells. AR-V7 was not detected in LNCaP cells. Bars show quantification normalized to the respective loading control (β-Actin). Superscript numbers indicate the membrane from which each loading control was derived, corresponding to the same membrane as the protein shown above.**d**, Western blot analysis showing protein expression of PSMA, ATGL, AR, AR-V7, and pAKT in LNCaP and 22RV1 cells treated with DMSO (control) or enzalutamide at IC25 (20 µM LNCaP, 22.5 µM 22RV1) and IC50 (40 µM LNCaP, 45 µM 22RV1) concentrations for 72 h. pAKT was not detected in 22RV1 cells. AR-V7 was not detected in LNCaP cells. Bars show quantification normalized to the respective loading control (β -Actin). Superscript numbers indicate the membrane from which each loading control was derived, corresponding to the same membrane as the protein shown above.

**Supplementary Figure S7:** Seahorse XF Mito Fuel Flex Test profiling under genetic silencing of ATGL and PSMA in LNCaP and 22RV1 cells

Energy maps (**a**,**c**) display OCR plotted against ECAR for each individual well at the third baseline measurement before pathway inhibitor injection. Each dot represents one well irrespective of its subsequent measurement condition. Delta contribution plots (**b**,**d**) present the mean change in dependency (upper) and flexibility (lower) for glucose, glutamine, and fatty acid oxidation relative to the vehicle control (Lipofectamine). Error bars represent the propagated standard error of the mean. These assays were performed as single biological experiments with 1 to 4 technical replicate wells per measurement condition after quality control. **a**, Energy map for LNCaP cells following siRNA-mediated knockdown of ATGL (ATGL KD) or PSMA (PSMA KD). **b**, Delta contribution plots for LNCaP knockdown cells relative to Lipofectamine control. **c**, Energy map for 22RV1 cells following siRNA-mediated knockdown of ATGL or PSMA. **d**, Delta contribution plots for 22RV1 knockdown cells relative to Lipofectamine control.

**Supplementary Table 1:** Clinicopathological characteristics for the patient cohort used in the discovery proteomics and RRBS analyses.

**Supplementary Table 2:** Clinicopathological characteristics for the independent validation cohort.

**Supplementary Dataset 1:** Complete results of the differential protein expression analysis comparing PSMA-high versus PSMA-low tumour regions

**Supplementary Dataset 2:** Complete results of the differential methylation analysis from RRBS data, comparing paired PSMA-high versus PSMA-low tumour regions. Includes genomic coordinates of differentially methylated regions and associated statistical values.
